# A combined microscopy and single-cell sequencing approach reveals the ecology, morphology, and phylogeny of uncultured lineages of zoosporic fungi

**DOI:** 10.1128/mbio.01313-23

**Published:** 2023-07-24

**Authors:** Kensuke Seto, D. Rabern Simmons, C. Alisha Quandt, Thijs Frenken, Alden C. Dirks, Rebecca A. Clemons, Katelyn M. McKindles, R. Michael L. McKay, Timothy Y. James

**Affiliations:** 1 Department of Ecology and Evolutionary Biology, University of Michigan, Ann Arbor, Michigan, USA; 2 Faculty of Environment and Information Sciences, Yokohama National University, Yokohama, Kanagawa, Japan; 3 Department of Botany and Plant Pathology, Purdue University, West Lafayette, Indiana, USA; 4 Department of Ecology and Evolutionary Biology, University of Colorado Boulder, Boulder, Colorado, USA; 5 Great Lakes Institute for Environmental Research, University of Windsor, Windsor, Ontario, Canada; 6 Cluster Nature and Society, HAS University of Applied Sciences, 's-Hertogenbosch, the Netherlands; 7 Department of Integrative Biology, The University of Texas at Austin, Austin, Texas, USA; 8 Great Lakes Center for Fresh Waters and Human Health, Bowling Green State University, Bowling Green, Ohio, USA; Johns Hopkins Bloomberg School of Public Health, Baltimore, Maryland, USA

**Keywords:** early diverging fungi, parasite, phylogeny, single-cell analysis

## Abstract

Environmental DNA analyses of fungal communities typically reveal a much larger diversity than can be ascribed to known species. Much of this hidden diversity lies within undescribed fungal lineages, especially the early diverging fungi (EDF). Although these EDF often represent new lineages even at the phylum level, they have never been cultured, making their morphology and ecology uncertain. One of the methods to characterize these uncultured fungi is a single-cell DNA sequencing approach. In this study, we established a large data set of single-cell sequences of EDF by manually isolating and photographing parasitic fungi on various hosts such as algae, protists, and micro-invertebrates, combined with subsequent long-read sequencing of the ribosomal DNA locus (rDNA). We successfully obtained rDNA sequences of 127 parasitic fungal cells, which clustered into 71 phylogenetic lineages belonging to seven phylum-level clades of EDF: Blastocladiomycota, Chytridiomycota, Aphelidiomycota, Rozellomycota, and three unknown phylum-level clades. Most of our single cells yielded novel sequences distinguished from both described taxa and existing metabarcoding data, indicating an expansive and hidden diversity of parasitic taxa of EDF. We also revealed an unexpected diversity of endobiotic *Olpidium*-like chytrids and hyper-parasitic lineages. Overall, by combining photographs of parasitic fungi with phylogenetic analyses, we were able to better understand the ecological function and morphology of many of the branches on the fungal tree of life known only from DNA sequences.

**IMPORTANCE:**

Much of the diversity of microbes from natural habitats, such as soil and freshwater, comprise species and lineages that have never been isolated into pure culture. In part, this stems from a bias of culturing in favor of saprotrophic microbes over the myriad symbiotic ones that include parasitic and mutualistic relationships with other taxa. In the present study, we aimed to shed light on the ecological function and morphology of the many undescribed lineages of aquatic fungi by individually isolating and sequencing molecular barcodes from 127 cells of host-associated fungi using single-cell sequencing. By adding these sequences and their photographs into the fungal tree, we were able to understand the morphology of reproductive and vegetative structures of these novel fungi and to provide a hypothesized ecological function for them. These individual host-fungal cells revealed themselves to be complex environments despite their small size; numerous samples were hyper-parasitized with other zoosporic fungal lineages such as Rozellomycota.

## INTRODUCTION

Estimates on the number and diversity of fungi have been radically altered by the widespread adoption of culture-independent methods, such as metabarcoding and metagenomics (1 - 3). These studies highlight the gap between the formally described fungal taxa and the estimated diversity, suggesting perhaps only 5%–10% of all fungal species have been described (4, 5). Moreover, they often identify major gaps in our knowledge of fungal phylogeny, such as entirely new lineages of fungi that were previously undetected (6 - 9). As novel as these sequence-based discoveries can be, one of the major hurdles to really understanding fungal diversity is a phenotypic characterization of the novel fungal lineages that comprise the so-called dark matter fungi found in metabarcoding studies (10). One approach to breaking through this barrier is the development of single-cell sequencing methods that rely on direct observations of cells through microscopy that can then be isolated and subjected to DNA sequencing and phylogenetic comparison to novel lineages from environmental DNA surveys (11 - 15). This way, information on both the habitat (e.g., host or substrate) and morphology can be obtained for these dark matter lineages.

Single-cell methods are particularly appropriate for studying the early diverging fungi (EDF), which are primarily microscopic and often unicellular. Metabarcoding studies show that many habitats are rich in novel EDF (8, 9, 16). Knowledge of the full diversity of EDF is growing, and new phyla are continuing to be described in this part of the tree (17 - 19). The fact that the undescribed EDF have never been cultured is likely because many of these fungi are parasitic (20 - 22). These fungi comprise a large portion of communities and are thus also ecologically relevant (16, 23 - 25). EDF are involved in ecosystem functions such as organic matter decomposition and nutrient cycling, making ecosystems more complex, and thus contribute to food web stability (26, 27). However, the morphology and ecological role of these EDF are speculative because they are recognized based only on environmental sequences. In this study, we endeavored to illuminate the morphology of uncultured EDF by isolating, photographing, and DNA sequencing parasitic fungi from several different types of freshwater habitats.

**Fig 1:**
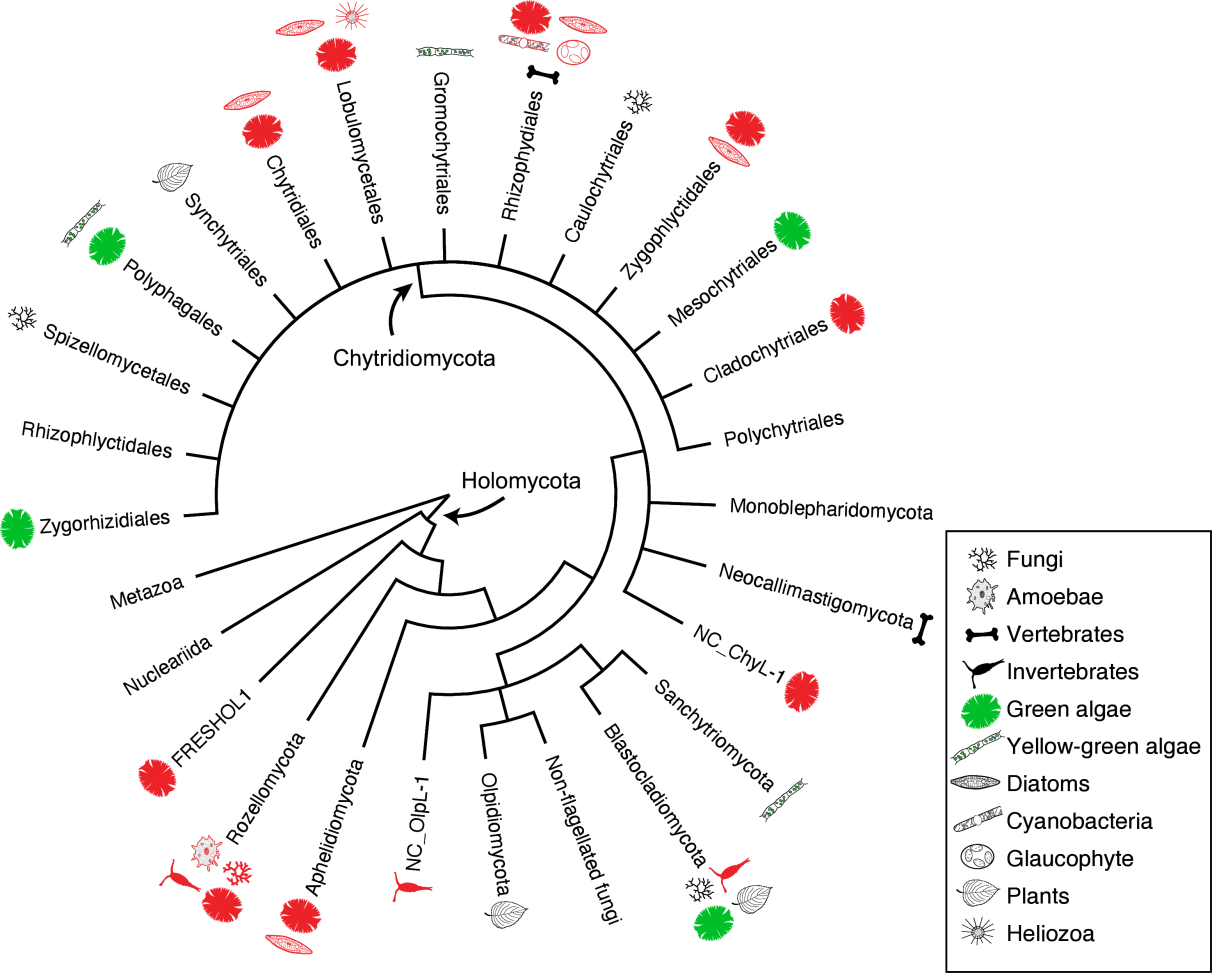
Schematic tree showing the phylogenetic relationships among the early diverging fungal phyla and orders in Chytridiomycota and their host range. Illustrations of each lineage indicate hosts of parasitic taxa. Red colored illustrations indicate hosts of single cells isolated in this study.

Our data fill in gaps in the constantly improving phylogenetic overview of EDF, which in the last two decades has been dramatically changed by extensive molecular phylogenetic analyses. Chytridiomycota (so-called chytrids) was divided into four independent phyla, Blastocladiomycota, Neocallimastigomycota, Monoblepharidomycota, and Chytridiomycota *sensu stricto* (28 - 30), plus the recent addition of phyla Olpidiomycota (18, 31) and Sanchytriomycota (17) (Fig. 1). In addition to chytrids *sensu lato*, Aphelidiomycota (=Aphelida, so-called aphelids, endoparasites of algae) and Rozellomycota (=Cryptomycota, so-called rozellids, endoparasites of fungi, animals, and protists) were recognized as the most basal lineages of fungi along with Microsporidia (5, 31). In some classifications, aphelids, rozellids, and Microsporidia have been treated as sister lineages of the true fungi because of the absence of a cell wall during the trophic phase and the presence of a phagotrophic nutrient strategy (although Microsporidia lack this feature) (22, 32, 33). The phylogeny and taxonomy of chytrids *sensu lato* have in the last two decades been biased toward culture-based observations and analyses mainly on saprotrophic chytrids (34). Parasitic chytrids as well as aphelids and rozellids can also be investigated by culture-based studies in which a parasite and its host are cultivated together and incorporated into phylogenetic analyses (20, 21, 35, 36), and these data have been vital for understanding host range across chytrid orders (Fig. 1). These culture-based studies have shown that algal parasites often represent new orders, families, or genera (20, 21, 37 - 41). Importantly, the orders Mesochytriales (20) and Zygophlyctidales (21) brought into formal definition novel clades that had previously only been known from environmental sequences (23, 42). Although further investigation of parasitic taxa is important to clarify the diversity of EDF, culture-based studies of parasitic taxa are difficult and time-consuming. Single-cell sequencing approaches can overcome some of these challenges and can be scaled up to higher throughput (11, 15).

In this study, a single-cell isolation approach was employed along with long-read sequencing techniques (34, 43) to comprehensively isolate parasitic EDF on various hosts and determine their phylogenetic position based on ribosomal DNA (rDNA) sequences. Sequence data for 127 parasitic fungal cells were successfully obtained and revealed 71 lineages, many of which were phylogenetically distinguished from described taxa. Additionally, single-cell lineages were compared with long-read metabarcoding data from similar habitats (44) to assess the overlap between culture-independent methods.

## RESULTS

### Phylogenetic position of isolated single cells

**Fig 2:**
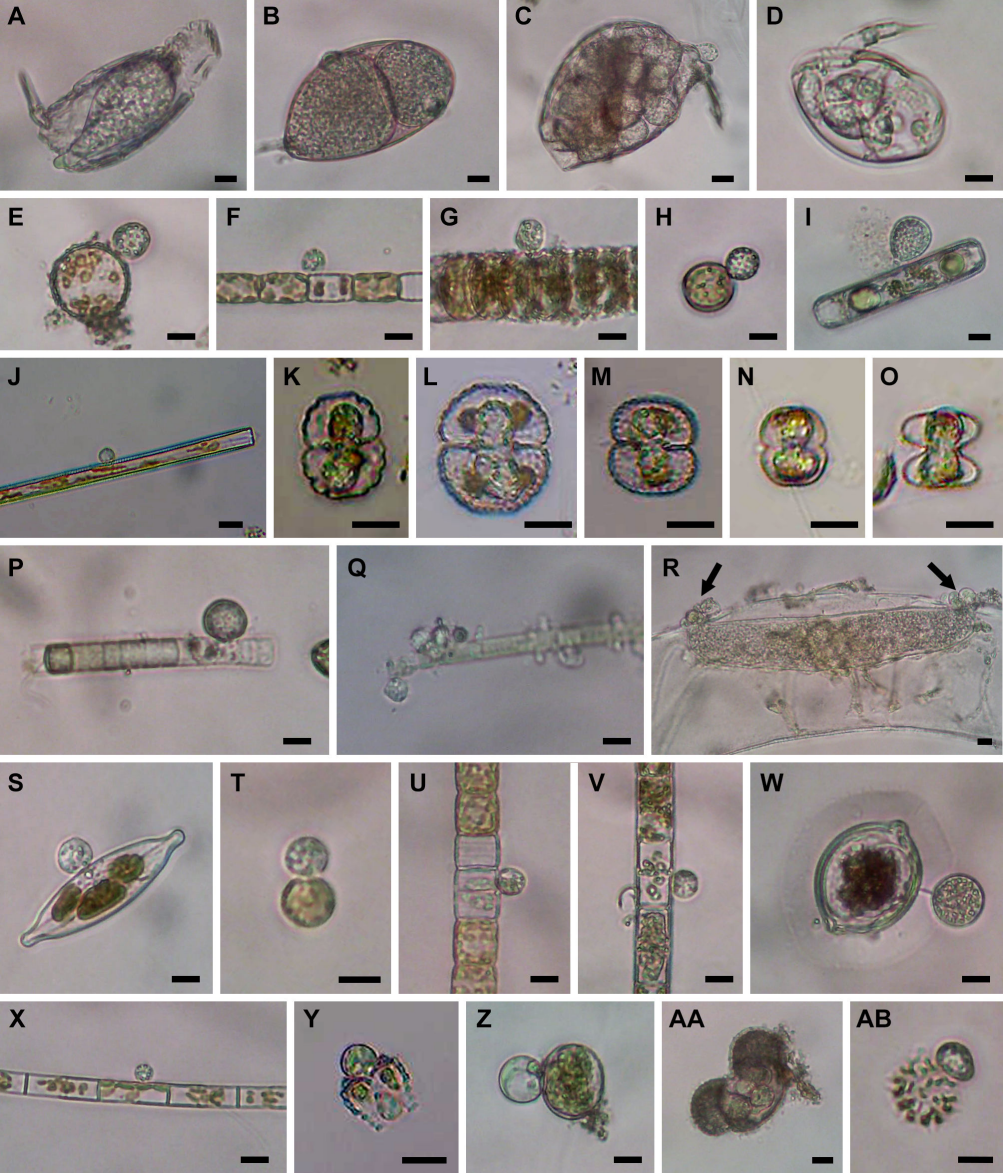
Microscopic images of isolated cells. (**A**) *Olpidium*-like chytrid PSC-L1 in rotifer. (**B**) *Olpidium*-like chytrid PSC-L2 in rotifer egg. (**C**) *Olpidium*-like chytrid PSC-L3 in rotifer. (**D**) *Olpidium*-like chytrid PSC-L4 in rotifer. (E–G) Chytrids PSC-L5 on *Stephanodiscus* spp. (**E and G**) and *Stephanodiscus binderanus* (**F**). (**H**) Chytrid PSC-L6 on *Stephanodiscus* sp. (**I**) Chytrid PSC-L7 on *Pinnularia* sp. (**J**) Chytrid PSC-L8 on *Ulnaria* sp. (K–O) *Olpidium*-like chytrids PSC-L9 in *Cosmarium* spp. (K–N) and *Staurastrum* sp. (**O**). (**P and Q**) Chytrid PSC-L10 on Oscillatoriales spp. (**R**) Hyper-parasitic chytrid PSC-L11 (arrows) attaching on elongated oomycete zoosporangium inside *Spirogyra* sp. (**S**) Chytrid PSC-L12 on *Craticula* sp. (**T**) Chytrid PSC-L13 on *Conticribra* sp. (**U**) Chytrid PSC-L14 on *Stephanodiscus binderanus*. (**V**) Chytrid on PSC-L15 on *Aulacoseira* sp. (**W**) Chytrid PSC-L16 on *Desmidium* sp. (**X**) Chytrid PSC-L17 on *Aulacoseira* sp. (**Y**) Chytrid PSC-L18 on *Staurastrum* sp. (**Z**) Chytrid PSC-L19 on *Glaucocystis* sp. (AA) *Olpidium*-like chytrid PSC-L20 in pine pollen. (AB) Chytrid PSC-L21 on *Stauridium* sp. All scale bars are 10 µm.

**Fig 3:**
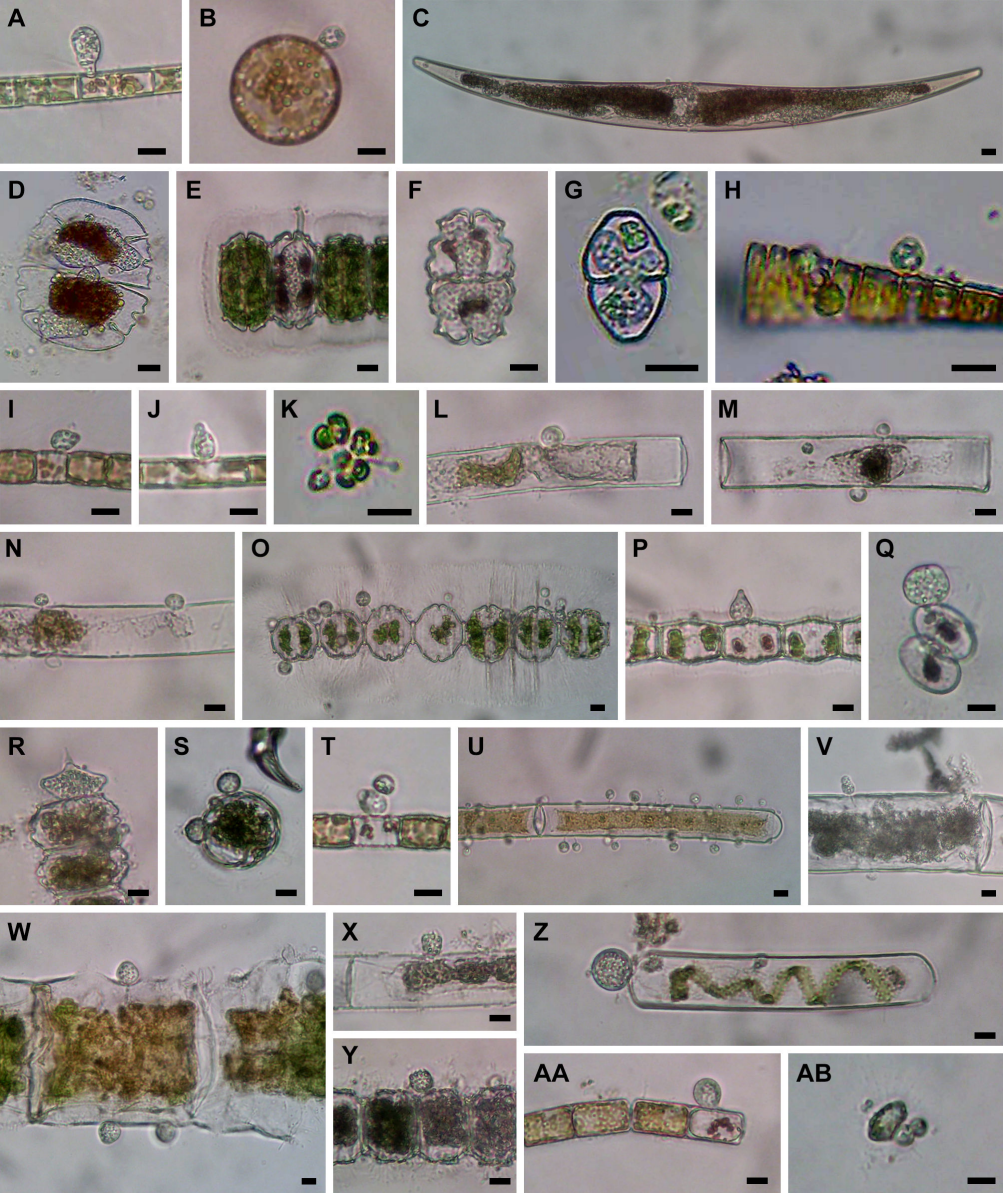
Microscopic images of isolated cells. (**A and B**) Chytrid PSC-L22 on *Aulacoseira* sp. (**A**) and *Stephanodiscus* sp. (**B**). (**C**) *Olpidium*-like chytrid PSC-L25 in *Closterium* sp. (**D**) *Olpidium*-like chytrid PSC-L23 in *Micrasterias truncata*. (**E**) *Olpidium*-like chytrid PSC-L24 in *Desmidium* sp. (**F**) *Olpidium*-like chytrid PSC-L26 in *Euastrum* sp. (**G**) *Olpidium*-like chytrid PSC-L27 in *Cosmarium* sp. (**H**) Chytrid PSC-L29 on *Fragilaria* sp. (**I**) Chytrid PSC-L28 on *Stephanodiscus binderanus*. (**J**) Chytrid PSC-L30 on *Aulacoseira ambigua*. (**K**) Chytrid PSC-L31 on *Sphaerocystis* sp. (**L**) Chytrid PSC-L32 on *Mougeotia* sp. (**M**) Chytrid PSC-L33 on *Mougeotia* sp. (**N**) Two chytrids PSC-L32 and L33 on *Mougeotia* sp. (**O**) Chytrid PSC-L34 on *Desmidium* sp. (**P**) Chytrid PSC-L35 on *Bambusina* sp. (**Q**) Chytrid PSC-L36 on *Cosmarium* sp. (**R**) Chytrid on *Desmidium* sp. (**S**) Chytrid PSC-L38 on *Desmidium* sp. (**T**) Hyper-parasitic chytrid PSC-L39 attaching on another chytrid on *Stephanodiscus binderanus*. (**U**) Chytrid PSC-L40 on *Mougeotia* sp. (**V**) Chytrid PSC-L41 on *Spirogyra* sp. (**W**) Chytrid PSC-L42 on *Spirogyra* sp. (**X**) Chytrid PSC-L43 on *Mougeotia* sp. (**Y**) Chytrid PSC-L44 on *Desmidium* sp. (**Z**) Chytrid PSC-L45 on *Spirogyra* sp. (AA) Chytrid PSC-L46 on *Melosira varians*. (AB) Chytrid PSC-L47 on unidentified heliozoan. All scale bars are 10 µm.

**Fig 4:**
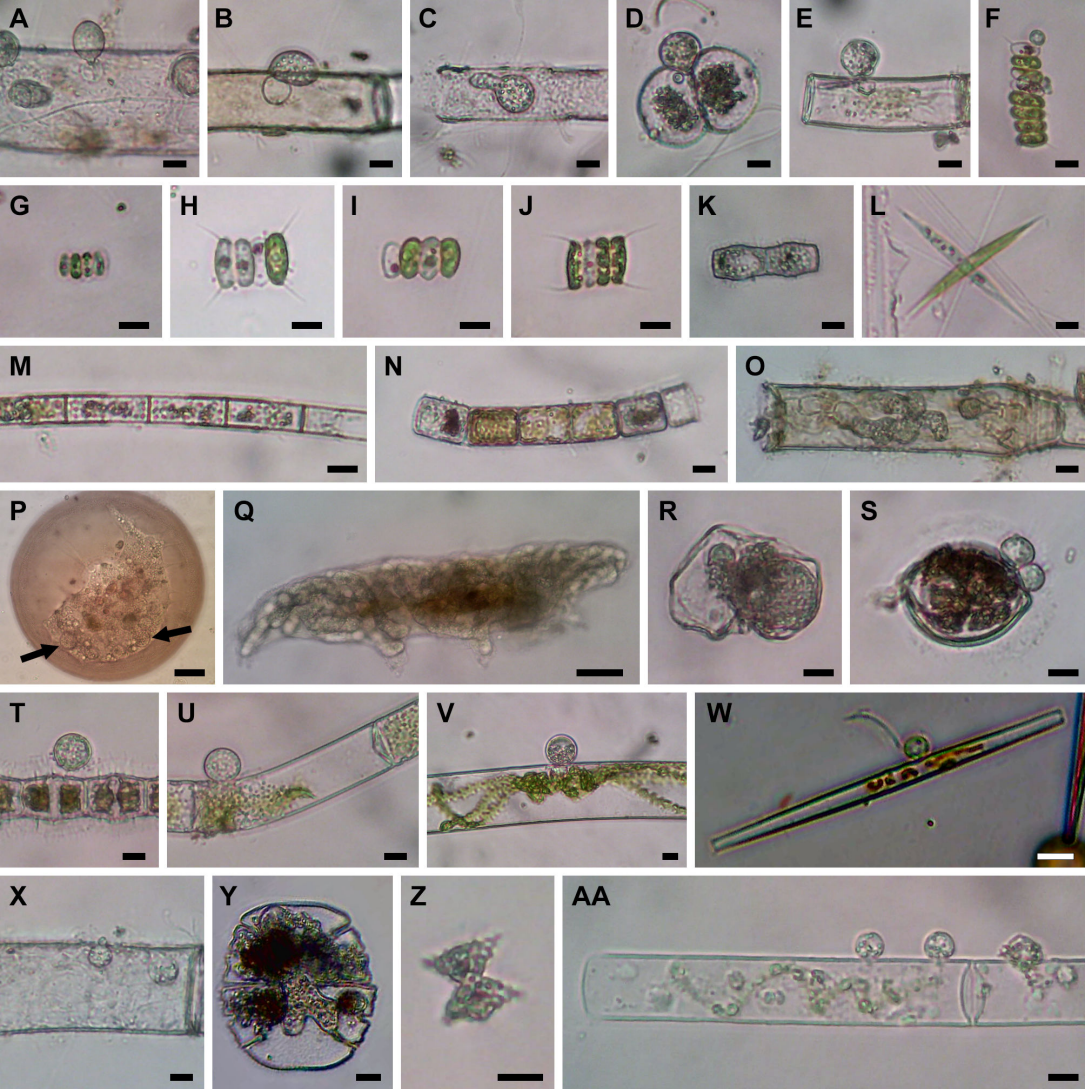
Microscopic images of isolated cells. (**A**) Chytrid PSC-L48 on *Oedogonium* sp. (**B**) Chytrid PSC-L49 on *Oedogonium* sp. (**C**) Chytrid PSC-L50 in *Oedogonium* sp. (**D and E**) Chytrid PSC-L51 on *Cosmarium* sp. (**D**) and *Oedogonium* sp. (**E**). (**F**) Chytrid PSC-L52 on *Desmodesmus* sp. (**G and H**) Aphelid PSC-L53 in *Scenedesmus* sp. (**G**) and *Desmodesmus* sp. (**H**). (**I and J**) Aphelid PSC-L54 in *Scenedesmus* sp. (**I**) and *Desmodesmus* sp. (**J**). (**K**) Two aphelids PSC-L55 and L59 in *Bambusina* sp. (**L**) Aphelid PSC-L58 in *Ankistrodesmus* sp. (**M**) Aphelid PSC-L56 in *Aulacoseira* sp. (**N**) Aphelid PSC-L57 in *Melosira varians*. (**O**) Isolated cell of rozellid PSC-L60 including *Oedogonium* sp. and endobiotic, tube-shaped zoosporangia. (**P**) Microsporidia-like rozellid PSC-L61 (indicated by arrows) in *Arcella* sp. (**Q**) Isolated cell of rozellids PSC-L62 including tardigrade and tube-shaped zoosporangia. (**R**) Isolated cell of rozellids PSC-L63 including putative broken rotifer body and endobiotic zoosporangium. (S−Y) Hyper-parasitic *Rozella* infecting parasitic chytrids: PSC-L64 in chytrids on *Desmidium* sp. (**S**) and *Bambusina* sp. (**T**), PSC-L65 in chytrid on *Mougeotia* sp. (**U**), PSC-L66 in chytrid on *Spirogyra* sp. (**V**), PSC-L67 in chytrid on *Ulnaria* sp. (**W**), PSC-L68 in chytrid in *Oedogonium* sp. (**X**), and PSC-L69 in *Olpidium*-like chytrid in *Micrasterias truncata* (**Y**). (**Z**) *Staurastrum* sp. harboring unknown fungus PSC-L70. (AA) Isolated cell of unknown fungus PSC-L71 including *Spirogyra* sp. and attaching chytrid-like sporangia.

**Fig 5:**
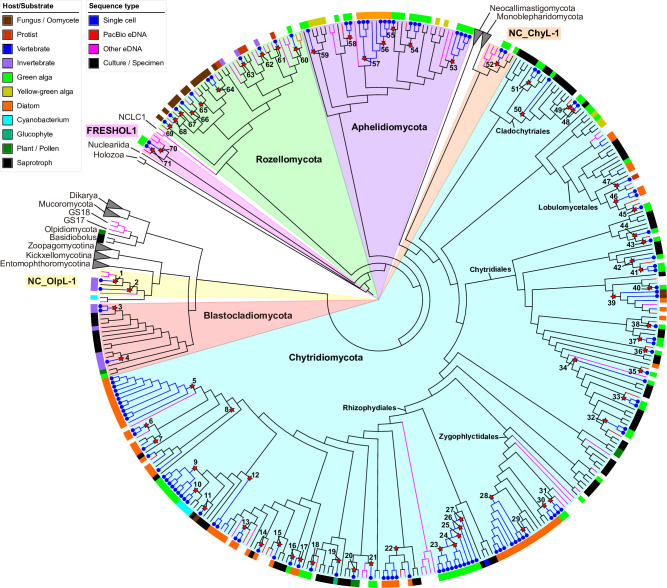
Maximum likelihood (ML) tree of 18S-5.8S-28S rDNA concatenated data set. Outer ring indicates the host/substrate of each culture or single cell. Brach color indicates sequence types (single cell, environmental DNA, PacBio OTU in this study, or culture/specimen). Blue circles on the tips indicate single-cell sequences obtained in this study. Red stars on the nodes indicate single-cell lineages reported in this study and the numbers correspond to the lineage numbers in the text (PSC-L1–71).

**Fig 6:**
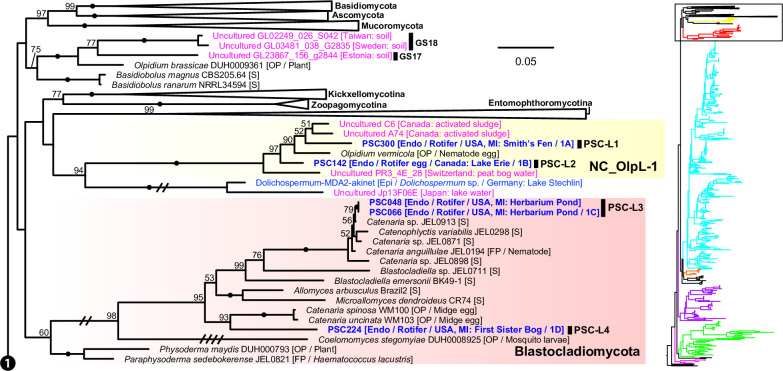
Portion of maximum likelihood (ML) tree of 18S-5.8S-28S rDNA concatenated data set including Ascomycota, Basidiomycota, Mucoromycota, Entomopthoromycotina, Kickxellomycotina, Zoopagomycotina, Blastocladiomycota, and the NC_OlpL-1 clade. ML bootstrap values higher than 50% were shown on each branch. Black dots on branches indicate 100% bootstrap value. Double and quadruple slashes on branches indicate that length is reduced by half and quarter, respectively. Cultured fungi are labeled in black; saprotrophs are indicated as [S], and obligate [OP] and facultative [FP] parasites are indicated as [O(F)P / its host]. Single cells isolated in this study are labeled in bold blue and previously published sequences of single cells are labeled in blue; annotations are indicated as [Endo (endobiotic) or Epi (epibiotic) / host / isolation source / figure number if available]. Published environmental DNA sequences are labeled in pink and PacBio OTU sequences in this study are labeled in bold red; source of each sequence is described in parentheses.

**Fig 7:**
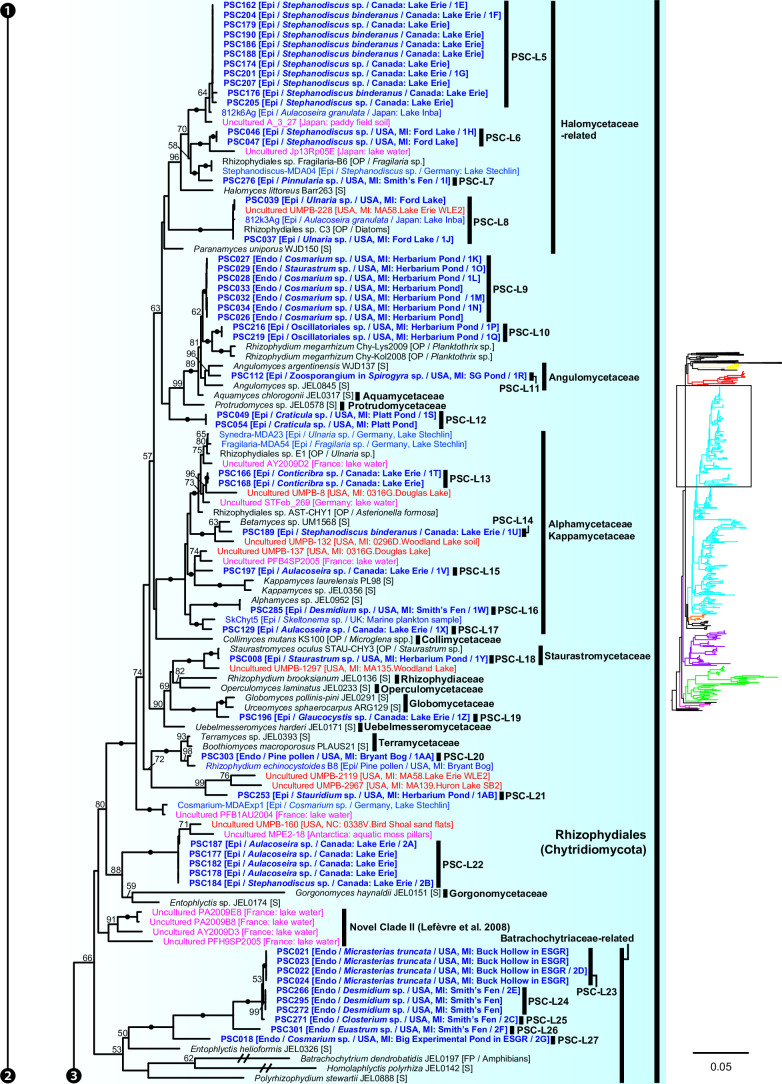
Portion of maximum likelihood (ML) tree of 18S-5.8S-28S rDNA concatenated data set including order Rhizophydiales in Chytridiomycota.

**Fig 8:**
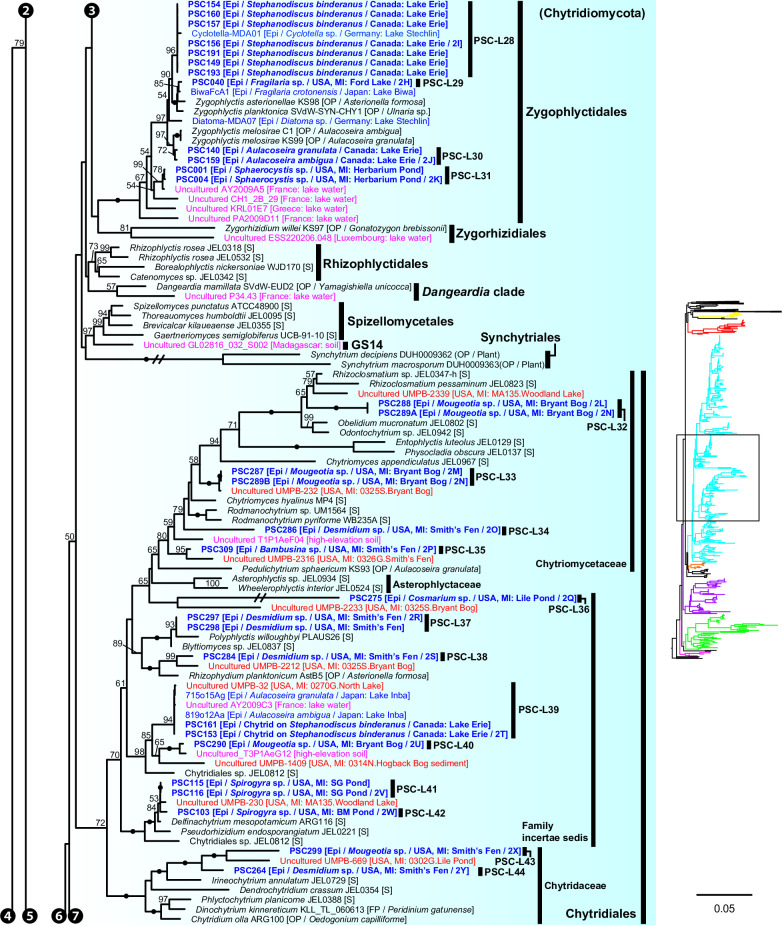
Portion of maximum likelihood (ML) tree of 18S-5.8S-28S rDNA concatenated data set including orders Zygophlyctidales, Zygorhizidiales, Rhizophlyctidales, Spizellomycetales, Synchytriales, and Chytridiales in Chytridiomycota.

**Fig 9:**
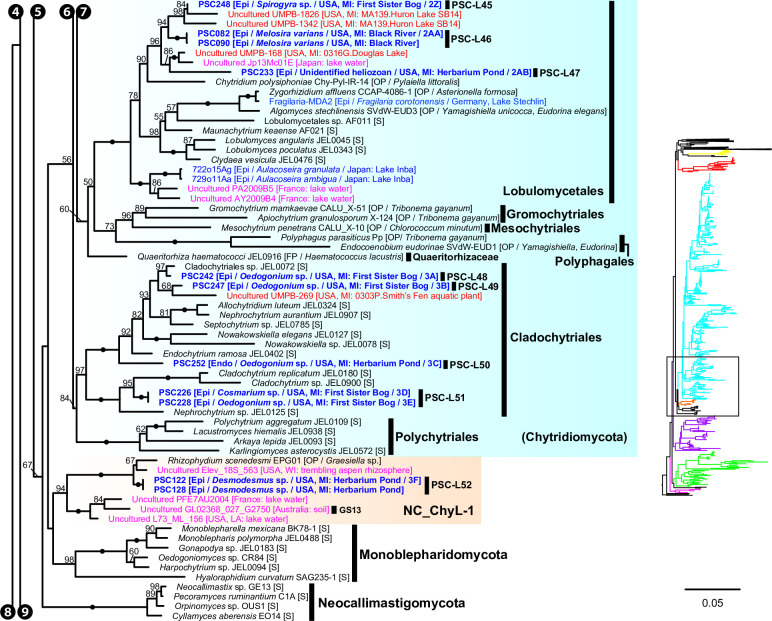
Portion of maximum likelihood (ML) tree of 18S-5.8S-28S rDNA concatenated data set including Monoblepharidomycota, Neocallimastigomycota, the NC_ChyL-1 clade, and orders Lobulomycetales, Gromochytriales, Mesochytriales, Polyphagales, Cladochytrilaes, and Polychytriales in Chytridiomycota.

**Fig 10:**
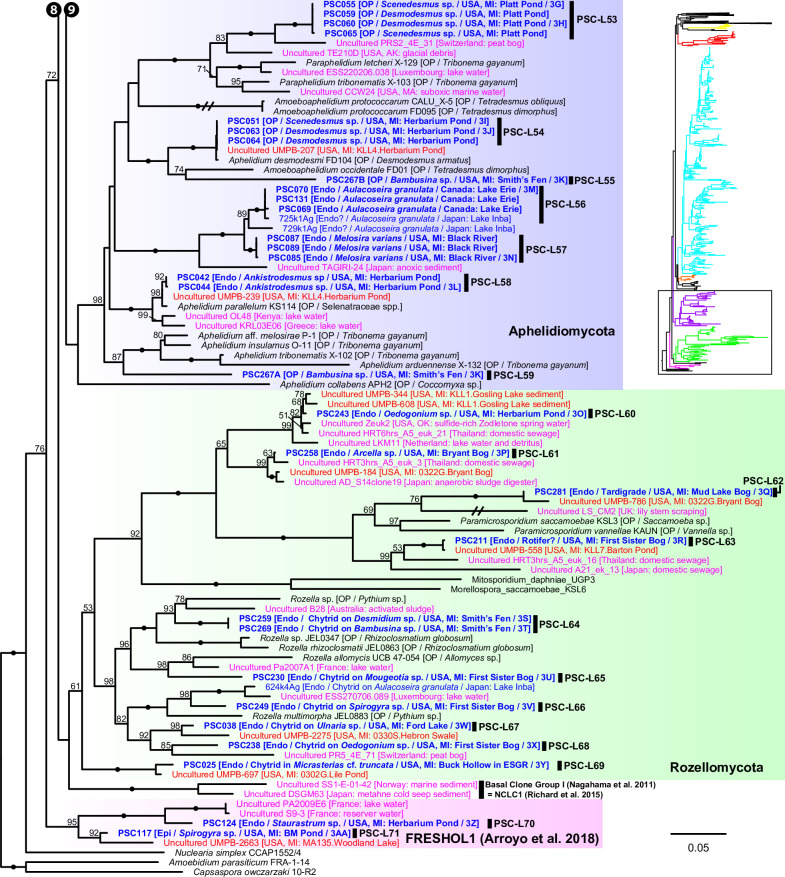
Portion of maximum likelihood (ML) tree of 18S-5.8S-28S rDNA concatenated data set including Aphelidiomycota, Rozellomycota, the NCLC1 and FRESHOL1 clade, *Nuclearia simplex*, and outgroup taxa (two holozoan taxa).

Over 300 individual cells of chytrid-, aphelid-, and Microsporidia-like fungi associated with their various hosts such as green algae, diatoms, cyanobacteria, protists, and micro-invertebrates were isolated (Fig. 2 to 4). A single-cell pipeline was applied to 259 isolated cells (excluding some duplicated samples and putative non-fungal cells such as oomycetes and cercozoans, data not shown), and fungal rDNA sequences were successfully obtained for 129 cells by the Oxford Nanopore Technologies (ONT) or Sanger method (see Table S1 in the supplemental material). Excluding the two zygomycetous sequences (PSC016 and PSC279, Table S1), 127 sequences were used for subsequent analyses. Based on the phylogenetic analysis on the concatenated data set of 18S-5.8S-28S rDNA sequences (Fig. 5 to 10, full tree along with the photos of isolated cells is available as “pursuit_tree.html” at Deep Blue repository, https://dx.doi.org/10.7302/7000), the 127 cells were categorized into 71 lineages distributed among seven phylum-level clades of EDF: Blastocladiomycota, Chytridiomycota, Aphelidiomycota, Rozellomycota, and three clades of unknown phyla.

We found five single-cell lineages that could not be placed into any phylum (i.e., phylum *incertae sedis*) (Fig. 5). Two lineages were *Olpidium*-like endoparasites of adult rotifers (PSC-L1; Fig. 2A) and rotifer eggs (PSC-L2; Fig. 2B) and formed a novel clade named NC_OlpL-1 (Novel Clade of *Olpidium*-like-1, Fig. 6). This clade also includes three environmental sequences and the unpublished sequence data of *Olpidium vermicola*. PSC-L52, an epibiotic chytrid on *Desmodesmus* sp. (Fig. 4F), was related to *Rhizophydium scenedesmi* strain EPG01 on *Grasiella* sp. (45). Along with some environmental sequences, these chytrids formed a distinct clade named NC_ChyL-1 (Novel Clade of Chytrid-like-1, Fig. 9), which is sister to Monoblepharidomycota, but statistical support for this relationship was not robust. Two lineages were placed in the clade FRESHOL1 reported previously (6) (Fig. 10). PSC-L70 was the cell of *Staurastrum* sp. filled by uncolored particles (Fig. 4Z). PSC-L71 was epibiotic chytrid-like cells on *Spirogyra* sp. (Fig. 4AA).

Only two lineages, both endobiotic parasites of adult rotifers, were placed in the Blastocladiomycota (Fig. 5 and 6). PSC-L3 (Fig. 2C) was placed in the clade including *Catenaria anguillulae* and *Catenophlyctis variabilis*. PSC-L4 (Fig. 2D) was sister to two *Catenaria* spp. parasitic on midge eggs (46, 47).

Most (*n* = 47) lineages were placed in Chytridiomycota, distributed among five orders: Rhizophydiales, Zygophlyctidales, Chytridiales, Lobulomycetales, and Cladochytriales (Fig. 5). Rhizophydiales was the most abundant in our isolates including 23 lineages (Fig. 7) on various hosts or substrates: 10 on diatoms (PSC-L5–8, 12–15, 17, 22; Fig. 2E through J, S through V, X, and 3A and B), 8 on zygnematophycean green algae (PSC-L9, 16, 18, 23–27; Fig. 2K through O, W, Y and 3C through G), and 1 each on chlorophycean green algae (PSC-L21; Fig. 2AB), glaucophyte algae (PSC-L19; Fig. 2Z), cyanobacteria (PSC-L10; Fig. 2P and Q), pine pollen (PSC-L20; Fig. 2AA), and oomycetes (PSC-L11; Fig. 2R). PSC-L11 was a putative hyper-parasitic chytrid attached to an endobiotic oomycete zoosporangium parasitizing *Spirogyra* sp. (Fig. 2R, arrows). Of the Rhizophydiales lineages, 16 exhibited typical epibiotic zoosporangium morphology, but the other seven were endobiotic zoosporangia in zygnematophycean green algae (PSC-L9, 23–27; Fig. 2K through O and 3C through G) or pine pollen (PSC-L20; Fig. 2AA). Most of our Rhizophydiales cells were distinguished from any cultivated chytrids, while three lineages (PSC-L8, 16, 18) were nearly identical to cultures of parasitic or saprotrophic chytrids.

In Zygophlyctidales (Fig. 8), three lineages of diatom parasites (PSC-L28–30; Fig. 3H through J) formed a clade along with known diatom parasitic species, *Zygophlyctis asterionellae*, *Z. planktonica*, and *Z. melosirae*. An additional lineage, PSC-L31, was parasitic on the green alga *Sphaerocystis* sp. (Fig. 3K) and closely related to the environmental sequence AY2009A5 from a lake in France (48).

In Chytridiales (Fig. 8), 13 lineages were found, 12 of which were epibiotic chytrids on zygnematophycean green algae such as *Bambusina* (PSC-L35; Fig. 3P), *Cosmarium* (PSC-L36; Fig. 3Q), *Desmidium* (PSC-L34, 37, 38, and 44; Fig. 3O, R, S and Y), *Mougeotia* (PSC-L32, 33, 40, and 43; Fig. 3L through N, U, and X), and *Spirogyra* (PSC-L41 and 42; Fig. 3V and W). Regarding the cell PSC289 (Fig. 3N), sequences of two independent lineages (PSC-L32 and 33) were obtained by ONT sequencing, indicating that two morphologically similar chytrids infected a single host. An additional lineage, PSC-L39 (Fig. 3T), was a putative hyper-parasitic chytrid on the Zygophlyctidales chytrid PSC-L28 on *S. binderanus* (see Discussion). Six lineages belonged to the known families Chytriomycetaceae (*n* = 4) and Chytridiaceae (*n* = 2), but they were distinct from any described taxa. Outside of these families, four additional lineages (PSC-L36, 38, 39, and 40) related to environmental sequences were found. In contrast, three lineages were closely related to described taxa. PSC-L37 (Fig. 3R) could be morphologically identified as *Polyphlyctis unispina* which was originally found from the same location as our isolates (49). This lineage was sister to another species of the genus, *P. willoughbyi*. PSC-L41 and 42 (Fig. 3V and W) were sister to the saprotrophic chytrid *Delfinachytrium mesopotamicum*.

In Lobulomycetales (Fig. 9), three lineages were found: PSC-L45 on *Spirogyra* sp. (Fig. 3Z), PSC-L46 on *Melosira varians* (Fig. 3AA), and PSC-L47 on an unidentified heliozoan (Fig. 3AB). These lineages were related to environmental sequences and separated from the core Lobulomycetaceae clade including the type genus *Lobulomyces*.

Three lineages of putative saprotrophs on dead green algae were placed in Cladochytriales (Fig. 9). PSC-L48 (Fig. 4A) and PSC-L49 (Fig. 4B) on *Oedogonium* spp. were characterized by an epibiotic zoosporangium with a conspicuous endobiotic apophysis, resembling described taxa such as *Chytridium lagenaria* and *C. schenkii* (50, 51). PSC-L50 (Fig. 4C) could be an endobiotic zoosporangium with a discharge tube inhabiting the cell of dead *Oedogonium* sp. PSC-L51 on *Cosmarium* sp. (Fig. 4D) and *Oedogonium* sp. (Fig. 4E) was sister to *Cladochytrium* spp.

We found seven lineages in Aphelidiomycota (Fig. 10). Aphelid cells were recognizable based on host algal cells filled by a parasite cell with a conspicuous, red-colored residual body (Fig. 4G through N). Two lineages were parasitic on *Desmodesmus* and *Scenedesmus* and separated into independent clades: PSC-L53 (Fig. 4G and H) was placed in the clade including *Paraphelidium* spp. on *Tribonema gayanum* (52, 53) and PSC-L54 (Fig. 4I and J) was nearly identical to *Aphelidium desmodesmi* on *Desmodesmus armatus* (54). Similar to the cell PSC289 in Chytridiales, PSC267 on *Bambusina* sp. (Fig. 4K) included two distinct lineages: PSC-L55 sister to *Amoeboaphelidium occidentale* on *Scenedesmus dimorphus* (55, 56) and PSC-L59 sister to *Aphelidium* spp. parasitic on *T. gayanum* (57 - 60). Two lineages of diatom parasites, PSC-L55 on *A. granulata* (Fig. 4M) and PSC-L56 on *M. varians* (Fig. 4N), were distinct from described species. PSC-L58 on *Ankistrodesmus* sp. (Fig. 4L) was closely related to *A. parallelum* parasitic on selenastracean green alga (61).

In Rozellomycota, 10 single-cell lineages were found (Fig. 10). Many of them were recognized as epibiotic or endobiotic zoosporangia on algae (Fig. 4O, S through Y) or micro-invertebrates (Fig. 4Q and R). PSC-L61 was a cell of *Arcella* sp. harboring a sac-like structure including Microsporidia-like spores (arrows in Fig. 4P). This appearance is similar to endoparasites of amoebae such as *Paramicrosporidium* (62) and *Morellospora* (63), which produce Microsporidia-like spores but are phylogenetically placed in Rozellomycota and much shorter branched than canonical Microsporidia. PSC-L61 was distinguished from these previously reported Microsporidia-like taxa. Five lineages (PSC-L64–68) were isolated as epibiotic chytrids on green algae (Fig. 4S through V, X) and diatoms (Fig. 4W). However, they were positioned in the *Rozella* clade, which comprises endoparasites of chytrids and oomycetes, suggesting they were hyper-parasites of chytrids. PSC-L69 (Fig. 4Y), which showed the same morphology as PSC-L23 (Fig. 3D) in Rhizophydiales, was sister to all other Rozellomycota taxa. This lineage is also a putative *Rozella*-like hyper-parasite (see Discussion). PSC-L60 was a tube-shaped zoosporangium in *Oedogonium* sp. (Fig. 4O) and placed in the LKM11 clade (64). PSC-L62 in a tardigrade (Fig. 4Q) and PSC-L63 in a putative broken rotifer body (Fig. 4R) were related to *Paramicrosporidium* spp. These zoosporangium-like structures in PSC-L60, L62, and L63 may not correspond to rozellids, and the sequences could be derived from hyper-parasites of these zoosporangia or undetected contaminated cells.

### Phylogenetic relationship between single cells and environmental sequences

The concatenated data set analysis showed that many of our single cells represented novel lineages distinguished from described taxa (Fig. 6 to 10). This result complements many environmental DNA studies that have reported unknown fungal lineages (23, 48). To examine overlap between our single-cell lineages and sequences only known from environmental DNA, we conducted a phylogenetic analysis on a comprehensive 18S rDNA data set including described taxa, environmental sequences available from NCBI database, with a focus on the phylum Chytridiomycota and phylum *incertae sedis* clades (see Fig. S1 in the supplemental material). In this analysis, new sequences of PacBio metabarcoding analyses primarily from Michigan, USA (44), were also used. Many of these PacBio sequences are derived from the same locations as the single cells isolated in this study, which gives a good opportunity to compare the two methods, metabarcoding and single-cell analysis, for exploring novel fungal diversity. The ML tree (see Fig. S1) showed that the vast majority of PacBio sequences represent entirely new lineages. Only a few of the single-cell lineages were closely related to PacBio environmental sequences. The PSC-L8 clade, in Rhizophydiales, included sequence UMPB-228 from Ford Lake, the same place where the two diatom parasites were isolated. In Chytridiales, PSC-L39 included UMPB-32 detected from multiple freshwater environments including Lake Erie (see Data S1 in the supplemental material) where some of the single cells were isolated. This lineage was also found in lakes in Japan (15) and France (48). Sequence UMPB-232 was the most abundant in Bryant Bog (see Data S1) and was related to PSC-L33 isolated from the same location. Although overlap of lineages in the PacBio and single-cell data sets was low in terms of species, multiple lineages of single cells had as their most closely related sequence an OTU from the PacBio data set, e.g., PSC-L13 and 21 in Rhizophydiales; PSC-L33, 35, 38, 43, and 44 in Chytridiales; PSC-L45 and 46 in Lobulomycetales; PSC-L49 in Cladochytriales; and PSC-L71 in the FRESHOL1 clade (see Fig. S1). On the other hand, both Mesochytriales and Polyphagales were represented by multiple OTUs in the PacBio data set but were absent in the single-cell data. Despite these exceptions, the general pattern was one of significant overlap of taxonomic genera and families in these two culture-independent approaches.

## DISCUSSION

By utilizing single-cell techniques, 71 EDF lineages were sampled, many of which were newly recognized branches in the phylogeny of EDF, even at the phylum-level. The approach in the present study focused on targeting and sequencing individual EDF cells one at a time. Photographs of the isolated cells have implications for the ecology, morphology, and life cycle of these newly discovered EDF lineages. Using these data, we discuss the (i) ecological role of these uncultivated lineages, (ii) ecology and morphology of novel phylum-level clades, (iii) phylogenetic diversity of an enigmatic chytrid genus *Olpidium*, and (iv) unexpected recovery of hyper-parasitic lineages. Finally, technical advances and challenges of single-cell approaches used in this study are also discussed.

### Shedding light on the ecological role of dark matter fungi

The cells isolated in this study were from diverse hosts, ranging from amoebae to invertebrates and especially algae. Some lineages are readily recognized as obligate algal parasites belonging to known parasite-specific groups such as Zygophlyctidales and Aphelidiomycota. Also found were many lineages of alga-associated cells in well studied orders such as Rhizophydiales, whose diversity has long been investigated based on numerous strains of saprotrophic taxa (65 - 67). Recently though several families of obligate algal parasites were described (37 - 68 - 68). Our data revealed further hidden diversity of putative parasitic lineages, representing new families or genera in the order. Although the isolated cells in this study were initially identified as “parasitic fungi,” some lineages we sampled in Chytridiales and Cladochytriales are putatively saprotrophic. When parasitic chytrids infect colonial algae, only dead cells are infected while living cells are uninfected (Fig. 2F, U, V and 3I, AA). In contrast, in some colonies, all algal cells are uncolored, or their chloroplasts are exhausted (Fig. 3O, P, R and Y), indicating the attaching chytrids grow on dead or moribund algae. Moreover, chytrids corresponding to PSC-L33 (Fig. 3M) in Chytridiales and PSC-L48 (Fig. 4A) in Cladochytriales were successfully isolated as pure cultures (data not shown).

These data also inform hypothetical ecological functions of lineages that were only known from metabarcoding approaches. For example, algal parasitic lineages PSC-L21 and 22 in Rhizophydiales formed independent clades along with some environmental sequences from aquatic environments, implicating a role for this clade as parasites of algae. Similarly, PSC-L45–47 in Lobulomycetales formed a novel clade including some environmental sequences from aquatic and soil environments, indicating that these lineages are parasites of algae and protists. Although Zygophlyctidales was previously thought to be composed only of diatom parasites (21), a lineage of a green algal parasite (PSC-L31) sister to an environmental sequence from a lake was found. This result indicates that other environmental sequence lineages in the order could exist as parasites of algae other than diatoms.

Generally, however, most of the single-cell sequences were poor matches at the species level to sequences from cultures or environmental DNA. This speaks to just how poorly we understand the true species level diversity of EDF, and how much work remains to be done in describing these fungi. In some cases, sequences from clades that were readily recovered with metabarcoding were not detected. The most striking case is Gromochytriales and Mesochytriales, together containing a total of three described species, all of which are obligate parasites (20, 69, 70). Despite limited described species, Mesochytriales is represented by numerous environmental DNA sequences (20). In this study, chytrids belonging to these orders were not identified with a single-cell approach. Instead, additional diversity was revealed based on phylogenetic analysis using PacBio metabarcoding data (see Fig. S1). Many of the sequences from lakes or ponds and some OTUs from Lake Erie, were related to *Mesochytrium penetrans*. This species is a parasite of a small green alga, *Chlorococcum minutum* (71), yet the collection strategy adopted for Lake Erie samples biased for larger colonial and filamentous forms. Further, most effort on Lake Erie was aligned with a winter science initiative (72), a season where diatoms are the dominant taxa associated with ice-cover in the lake (73). Thus, chytrids parasitic on smaller single cells or on taxa more prevalent during the summer may have been overlooked. More single-cell analyses on parasitic chytrids on various algae are necessary to reveal hidden taxa in the order.

While the pictures of the isolated cells can be informative in inferring their ecological role, sometimes they may be misleading. Specifically, each “cell” is actually a number of cells that include host, parasite, associated bacteria, and hyper-parasites. The latter were particularly common with some cells, such as PSC023, giving both an obvious chytrid pathogen as well as a likely *Rozella* hyper-parasite (74). Indeed, the majority of the Rozellomycota detected in this study were found as “by-catch” present in samples appearing as normal chytrids infecting algae (Fig. 4S through W). This result provides a slight cautionary tale that some of the sequences emerging from this approach may be most appropriately assigned not as parasites of the primary host but as hyper-parasites, an observation consistent with earlier results (15).

### Discovery of novel clades of early diverging fungi

Our approach was successful in revealing novel diversity at many taxonomic levels: species, genera, families, and even phyla. The novelty at the phylum level is consistent with recent phylogenetic analyses that have revealed that some parasitic fungi represent novel lineages worthy of phylum-level distinction (17, 18). We found and characterized three phylum *incertae sedis* clades (Fig. 5), two of which are newly reported in the present study. The NC_OlpL-1 clade includes two single-cell lineages of *Olpidium*-like chytrids on rotifers and *O. vermicola* parasitic on nematode eggs (75), indicating that this clade represents animal-associated endobiotic chytrids. NC_OlpL-1 was sister to a previously reported undescribed phylum-level clade represented by a single-cell isolate of a *Rhizosiphon*-like chytrid on the cyanobacterium *Dolichospermum* from a lake in Germany (11). In the tree by Van den Wyngaert et al. (11), *Dolichospermum* parasites were sister to the Chytridiomycota + Monoblepharidomycota + Neocallimastigomycota clade without strong statistical support. Although these putative novel phylum clades are related to Kickellomycotina, Zoopagomycotina, and Entomophthoromycotina in our tree (Fig. 6), the exact phylogenetic position is uncertain. Phylogenomic analysis would clarify the evolutionary history and taxonomy of these enigmatic lineages.

The NC_ChyL-1 clade, which included isolates of epibiotic chytrids on *Desmodesmus* (PSC-L52) and *Rhizophydium scenedesmi* strain EPG01 on *Graesiella* sp (45), was sister to Monoblepharidomycota without strong statistical support. Previously, *R. scenedesmi* was shown to be sister to the genus *Zygophlyctis* in Chytridiomycota (45). However, another analysis showed that *R. scenedesmi* along with some environmental sequences were placed sister to Monoblepharidomycota (21) as with the present study. This clade could correspond to the clade GS13 defined by Tedersoo et al. (8) because one of their environmental sequences (GL02368_027_G2750 from Australian soil) was positioned within NC_ChyL-1.

The clade FRESHOL1 was originally defined by Arroyo et al. (6) in their metabarcoding analysis of the Paraná River in Argentina. This clade was sister to all other fungi including Aphelidiomycota and Rozellomycota as with our analysis. PSC-L70 was a cell of *Staurastrum* sp. filled with a putative endoparasite (Fig. 4Z). The isolate corresponding to PSC-L71 included chytrid zoosporangium-like cells on *Spirogyra* sp. (Fig. 4AAA). Although information on the life cycles of these two lineages is currently limited, there is the possibility that they are endoparasites of algae or chytrid-like organisms in these samples. Two deep-branching groups of fungi, Aphelidiomycota and Rozellomycota, are known as endoparasites of other organisms (22). The previously defined phylum-level clade NCLC1 is sister to Rozellomycota in our tree (Fig. 10) and is also comprised of putative endoparasites of marine diatoms (76). Given the phylogenetic position and host of the FRESHOL1 lineage, our findings strengthen the recently suggested hypothesis that the ancestor of Fungi *sensu lato* (including aphelids, rozellids, microsporidians, and canonical fungi) had a symbiotic relationship with cellulose-based cell-walled taxa (77). Further observations and phylogenetic analyses of the FRESHOL1 clade are pivotal to elucidate the early evolution of Holomycota lineages.

### Phylogenetic diversity of *Olpidium*-like chytrids

We found *Olpidium*-like chytrids parasitic on various hosts such as adult rotifers (Fig. 2A, C, and D), rotifer eggs (Fig. 2B), desmid algae (Fig. 2K through O and 3C through G), and pine pollen (Fig. 2AA). The genus *Olpidium* is characterized by a holocarpic thallus, namely a simple thallus composed of only a zoosporangium without rhizoids (78). All species are endobiotic parasites of algae, plants, fungi, protists, and micro-invertebrates (78). Early molecular phylogenetic analyses (79, 80) revealed that plant root parasitic species are separated from core chytrid clades (e.g., Chytridiomycota and Blastocladiomycota) and related to zygomycetous fungi. A recent phylogenomic analysis showed that *O. bornovanus* parasitic on cucumber roots is sister to all terrestrial fungi (Dikarya + Mucoromycota + Zoopagomycota) (18). *Olpidium*-like chytrids obtained in the present study were not related to plant parasitic species (Olpidiomycota in Fig. 5) and were instead distributed among three other phylum-level clades.

Four lineages of rotifer parasites were placed in the NC_OlpL-1 clade (PSC-L1 and 2) and Blastocladiomycota (PSC-L3 and 4). The NC_OlpL-1 clade also included *O. vermicola* parasitic on nematode eggs. Apart from the plant parasitic lineage sister to Dikarya + zygomycetes (18), the NC_OlpL-1 clade is an additional putative independent phylum of *Olpidium*-like fungi. In Blastocladiomycota, PSC-L3 and 4 were related to taxa of the polyphyletic family Catenariaceae (81), which is characterized by polycentric thalli, consisting of catenulated zoosporangia connected by isthmuses (82). In both PSC-L3 and 4, multiple zoosporangia were seen in a single rotifer body but connections between zoosporangia were not visible. Some *Olpidium* species are known as rotifer parasites and often produce multiple zoosporangia in a single host, but early developmental stages have not been fully described (83 - 85). Some of these species could be related to Catenariaceae as with our rotifer parasites.

The other seven lineages of *Olpidium*-like chytrids were positioned in Rhizophydiales in Chytridiomycota (Fig. 7). Six of them were endoparasites of desmid algae: PSC-L9 related to Angulomycetaceae and PSC-L23–27 related to Batrachochytriaceae. PSC-L26 and 27 resemble *O. untricuriforme* in producing a branched tube-like zoosporangium (51). PSC-L24 on *Desmidium* sp. is similar to *O. hyalothecae* on *Hyalotheca dissiliens* (51); both infect algae of a filamentous clade in Desmidiaceae (86). Another lineage, PSC-L20 was an endobiotic chytrid in pine pollen and was related to Terramycetaceae. Rhizophydiales chytrids typically produce monocentric and epibiotic thalli with endobiotic rhizoidal systems (65). Exceptionally, *Batrachochytrium dendrobatidis* and *Entophlyctis helioformis* produce endobiotic thalli in amphibian skin cells and moribund green algal cells, respectively (87, 88). In *B. dendrobatidis*, rhizoids are rarely seen on zoosporangia in host skin in comparison to culture conditions (88). Simplification of thalli could occur easily in the endobiotic lifestyle. PSC-L23–27 were sister to *E. helioformis*, and these alga-associated endobiotic chytrids could be pivotal in investigating the evolution of nutritional modes and thallus morphology in Batrachochytriaceae.

Our phylogenetic analysis clearly showed that the genus *Olpidium* is polyphyletic, and that host generally tracks phylogeny. Tedersoo et al. (31) suggested accommodating *Olpidium* in the phylum Olpidiomycota based on the phylogenetic position of plant parasitic species of *Olpidium*. However, this taxonomic treatment should be examined by investigating more taxa, especially the type species, *O. endogenum*, which is known as a parasite on green algae of the genus *Closterium*.

### Unexpected findings of hyper-parasites

In the present study, we found putative hyper-parasites within Chytridiomycota and Rozellomycota. The two lineages in Chytridiomycota were clearly recognizable as a chytrid zoosporangium on top of another parasite. PSC-L11 in Rhizophydiales was parasitic on an elongated zoosporangium inside *Spirogyra* sp. (Fig. 2R). The host of this chytrid could be an endoparasitic oomycete in algae. Regarding similar described species, *Rhizophydium carpophilum* is known as a parasite of oogonia and oospores of *Saprolegnia* and *Achlya* (89) and also reported as a hyper-parasite of endoparasitic *Olpidiopsis* infecting *Achlya* (90). Another hyper-parasitic chytrid isolated in the present study is PSC-L39 in Chytridiales, a spherical zoosporangium on an epibiotic chytrid parasite on *S. binderanus* (Fig. 3T). Its host could be the chytrid of PSC-L28 (Fig. 3I) in Zygophlyctidales because the shape of the zoosporangium is similar, and they were found in the same sample collected at Lake Erie. Currently, some 15 species are known as epibiotic chytrid parasites of other chytrids (78, 91). One of them, *Septosperma anomalum,* was reported as a hyper-parasite infecting diatom parasite such as *Chytriomyces tabellariae* on *Tabellaria flocculosa* (92) and *Zygophlyctis asterionellae* on *Asterionella formosa* (93). Our isolates are distinguished from *S. anomalum* based on the shape of zoosporangium. Also, *S. anomalum* produces a unique resting spore with septation, which was not observed in our sample. Unfortunately, DNA sequence data are currently not available for any chytrid species parasitic on other chytrids, preventing comparison with our isolates. PSC-L39 corresponds to the clade CH_D including single-cell isolates from Lake Inba in Japan (15). These isolates were recorded as a chytrid parasite on *Aulacoseira* spp. but there is a possibility that its hyper-parasitic nature was overlooked.

The other single-cell lineages of hyper-parasites were found in Rozellomycota. These were isolated as epibiotic or endobiotic chytrid parasites of green algae or diatoms, but they were phylogenetically related to *Rozella* spp. (PSC-L64–69). The genus *Rozella* is well known as an endoparasite of chytrids or oomycetes (94). *Rozella* invades the host as an unwalled cell, consumes host cytoplasm by phagocytosis, and ultimately fills the entire host cell. Due to this endoparasitic nature, chytrid zoosporangia infected by *Rozella* might be difficult to detect, although some species cause hypertrophy or abnormal septation of the host cell (36, 95). Therefore, infections by *Rozella* were likely overlooked in our isolates. The putative hosts of our *Rozella* isolates were speculated: PSC-L64 (Fig. 4S) on Chytridiales chytrid PSC-L38 on *Desmidium* sp. (Fig. 3S); PSC-L66 (Fig. 4V) on Lobulomycetales chytrid PSC-L45 on *Spirogyra* sp. (Fig. 3Z); PSC-L67 (Fig. 4W) on Rhizophydiales chytrid PSC-L8 on *Ulnaria* sp. (Fig. 2J); and PSC-L69 (Fig. 4Y) on *Olpidium*-like chytrid PSC-L23 on *Micrasterias truncata* (Fig. 3D). Indeed, single-cell genomic analysis on the amplified genome of isolate PSC023 (lineage PSC-L23) revealed that the genome included both the host as well as the putative hyper-parasite corresponding to PSC-L69 (74). However, in our ONT sequencing, only a chytrid rDNA sequence was obtained in PSC023. We assume that biased PCR amplification occurred in this sample. Phylogenomic analysis showed that the Rozellomycota genome in PSC023 was sister to *Rozella* spp. although PSC-L69 separated from the *Rozella* clade in the present study. Our finding of hyper-parasitic *Rozella* indicates cryptic diversity of endoparasites infecting chytrids. These findings need to be taken into consideration when using single-cell approaches to infer nutritional mode from the recovered genomes.

### Technical advances and challenges using single-cell technique

The approach outlined presents both advances over traditional methods of single-cell genomics that involve fluorescence-activated cell sorting (96) as well as challenges. The primary advantages are that the method allows images of the target species to be obtained and that the success rate of going from cell to sequence is higher. Among the 259 cells processed with multiple displacement amplification (MDA), DNA sequence data were successfully generated for 139 cells (54%) in total. Excluding putative contaminants (e.g., cercozoans) and fungus-like organisms (e.g., oomycetes and hyphochytrids), 129 cells (50%) were categorized as fungal sequences (Table S1). This rate is higher than previous single-cell sequencing studies (single-cell sorting + whole genome amplification + PCR and sequencing) on planktonic prokaryotes and protists, which had a 5%–38% success rate (97 - 101). Moreover, photos accompanied these cells. While these cells are no longer available for morphological analysis, their amplified DNA with high concentration (147–1,600 ng/µL, Table S1) is present, which is facilitating ongoing genome sequencing. We believe that the high success rate of amplification and sequencing of the target cells is likely due to the fact that these fungal cells are actually comprised of multiple nuclei, in many cases representing the near mature reproductive stages of the chytrid zoosporangia that may contain 5–50 or more nuclei. A final technical advance is the combination of single-cell approaches with long rDNA PCR. Amplification of the majority of the coding bases of the rRNA operon in addition to the highly variable internal transcribed spacer region allows for robust phylogenetic placement as well as discrimination at the species level (102). Amplification of both 18S and 28S regions allows the data to be compared to multiple data sets, given that there are disparate uses of the two regions in both environmental DNA and systematics studies (48, 103).

There are also some disadvantages of the method. First, it is hard to scale up to a large number of cells because this is a manual approach in which each cell requires as much as an hour to find, clean, and pipette into a sample tube. Second, this approach requires considerable taxonomy, microscopy, and microbial natural history skills. These skills are lacking in most microbiology and mycology training. Third, there are biases in the targeting of hosts. Most of the isolated cells in this study were parasites of algae, although a few protists and micro-invertebrates were isolated. More diverse taxa could host parasitic EDF, but they may have been undersampled due to our limited ability to find them and diagnose them as infected. These biased isolations potentially hinder clarifying the diversity of EDF; such a limitation is less applicable to metabarcoding and metagenomic approaches. Finally, our samples are far from single cells, and often contain host cells, bacterial cells, and in several cases, hyper-parasites. This is both an advantage and disadvantage because it identifies interesting symbioses, but it also makes ascribing ecological function more complicated. Presence of host and bacterial DNA could limit the ability to sequence fungal genomes from these samples. In some cases, we were able to amplify host DNA in order to confirm species identity (data not shown), but in other cases, host DNA could not be recovered as presumably the parasite had already consumed it. Despite these disadvantages, the target single-cell isolation is a powerful method to investigate uncultured parasitic fungi, and its use will expand our understanding of the ecology and phylogeny of EDF.

## MATERIALS AND METHODS

### Sample collection and single-cell isolation

We collected 50–250 mL of water samples with detritus and/or plant material from ponds or lakes in Michigan in 2019–2021 (see Table S2 in the supplemental material). For Lake Erie, seston was collected by boat with a plankton net (≥20 µm) deployed 1–3 m from the surface, after which the collected material was transferred to a 50 mL conical centrifuge tube maintained at *in situ* water temperature in the dark. The samples were transferred to University of Michigan and incubated for ~1 month, at 20°C, under LED lighting. Water samples were observed using a Nikon TMS Inverted microscope (Nikon, Tokyo, Japan) to detect fungi associated with algae, micro-invertebrates, and protists. Detected fungal cells were photographed using Moticam X Camera (Motic, Hong Kong, China) or Dino-Eye Edge S Eyepiece Camera (AnMo Electronic Corporation, Taipei, Taiwan) digital cameras. Representative images were edited and assembled into plates using Adobe Photoshop. The cells were isolated manually using a manually prepared drawn-out glass capillary pipette. The isolated cells were washed by serial transfer in small drops (more than five) of UV-sterilized water, transferred into 200 µL PCR tubes with 1–2 µL of water, and kept at −80°C until DNA extraction.

### Whole genome amplification, PCR, and sequencing

To conduct DNA extraction and whole genome amplification of isolated cells by multiple displacement amplification (MDA), we used the Qiagen REPLI-g Single Cell Kit (Qiagen, Germantown, Maryland, USA) and processed the samples as described in Davis et al. (14). The DNA concentrations of some of the MDA products were measured with the Qubit 4 Fluorometer (Thermo Fisher Scientific, Waltham, MA, USA). To obtain rDNA sequences, we utilized the Oxford Nanopore Technology sequencing pipeline for zoosporic eufungi (34). To amplify the 18S-ITS1-5.8S-ITS2-28S rDNA operon, we performed long-range PCR with fungal specific primers, NS1short and RCA95m (43). We used TaKaRa LA Taq DNA polymerase (Takara Bio USA, San Jose, CA, USA) with the protocol described in Simmons et al. (34). For some samples, we used KOD Xtreme Hot Start DNA Polymerase (Merck Millipore, Burlington, MA, USA). We prepared 12.5 µL amplifications composed of the following: (i) 0.25 µL KOD extreme, (ii) 6.25 µL 2X Xtreme Buffer, (iii) 2.5 µL dNTPs, (iv) 0.75 µL each 5 µM barcoded primer NS1short/RCA95m, and (v) 2 µL 1/50 or 1/100 diluted MDA products. We performed PCR on an Eppendorf Mastercycler Pro S with the following conditions: (i) 95°C for 2 minutes, (ii) 10 cycles of denaturation at 98°C for 10 minutes, annealing at 55–50°C (0.5°C decrease per cycle) for 30 seconds, and extension at 68°C for 5 minutes, (iii) 30 cycles of 98°C for 10 seconds, 50°C for 30 seconds, and 68°C for 5 minutes. The PCR products (4.5–6 kbp) were assessed by electrophoresis. We generated long-read sequences with an Oxford Nanopore Technologies MinION device and MinKNOW software (Oxford Nanopore Technologies, Oxford, United Kingdom). We prepared pooled barcoded amplicon libraries with the ONT Ligation Sequencing Kit (LSK-109), following the manufacturer’s protocol. We generated fast5 sequencing reads in MinKNOW that we base-called in Guppy (ONT). With the resulting fastq reads, we quality filtered (104) with NanoFilt (105) and converted them to fasta files with Seqtk (https://github.com/lh3/seqtk). We demultiplexed the pooled data with MiniBar (106), assembled sequences in Canu 1.9 (107) with defined cut-off criteria (34, 104), polished sequences with Medaka (https://github.com/nanoporetech/medaka), and removed barcodes to produce the final rDNA operon sequences in Geneious 9.1.7 (Biomatters, Auckland, New Zealand). For samples that failed the long-range PCR for ONT sequencing, we attempted short-range PCR using primers: SR1.5 (108)/AU4v2 (109) and CRYPTO2-2F (109)/AU4v2 for partial 18S, ITS5 (110)/RCA95m for ITS and partial 28S, and LR0R (111)/RCA95m for partial 28S. PCR products were purified using ExoSAP-IT (Thermo Fisher Scientific, USA). Sequencing analyses were performed with Genewiz sequencing service (NJ, USA) using the following primers: SR1.5, CRYPTO2-2F, NS4 (110), and AU4v2 for 18S, ITS3, ITS4, ITS5 (110) for ITS, and LR0R and LR5 (112) for 28S.

### Phylogenetic analysis

To clarify phylogenetic positions of single cell isolates, a phylogenetic analysis of a concatenated data set of 18S, 5.8S, and 28S rDNA sequences was performed (see Table S3 in the supplemental material). The 5.8S rDNA sequences were extracted from the data of ITS1-5.8S-ITS2 using ITSx (113). Sequences were aligned using MAFFT v7.487 (114) with the “L-INS-I” method, and the alignment was trimmed using trimAl (115) with the “gappyout” method. The maximum likelihood (ML) tree was inferred with IQ-TREE 2 (116). The best model of each alignment was examined using ModelFinder (117) implemented in the IQ-TREE 2. According to the corrected Akaike information criterion (AICc), GTR + F + R9, JC + R4, and TIM3 + F + R9 models were selected for 18S, 5.8S, and 28S, respectively. An ML analysis was run with a partitioned model (118). The branch supports were assessed with standard non-parametric bootstrap analysis (100 replicates). The tree was visualized with FigTree (https://github.com/rambaut/figtree) and edited with Adobe Illustrator.

### PacBio metabarcoding analysis

Single-cell sequence data were compared to a large collection of environmental DNA sequences utilizing a recently developed PacBio metabarcoding data set of 18S amplicons (44). These data are complementary because many of the sampling localities are shared (i.e., are from aquatic habitats in Michigan). For this analysis, we extracted reference sequences of 339 PacBio OTUs that were putatively identified as Chytridiomycota using taxonomic assignment in Qiime v1.9.1 (119) with BLAST (120) and a curated version of the SILVA database (121), which was amended to include more EDF including Aphelidiomycota and Rozellomycota (available here as “Updated_Silva_Cryptos_Aphelids.txt”: https://github.com/Michigan-Mycology/Lab-Code-and-Hacks/tree/master/Cryptomycota_ecology/Data_files/). We performed further manual curation of the sequences identified as Chytridiomycota for putative chimeras by phylogenetic analyses. We prepared a reference data set of 18S rDNA of cultured Chytridiomycota species, single-cell sequences from our and previous studies, and outgroup taxa (see Table S4 in the supplemental material). The sequences were aligned and trimmed as described above. All OTU sequences were divided into two parts, the former and latter ~650 bp nucleotides. The original and divided PacBio sequences were added into the reference alignment using MAFFT with the “--add” and “--keeplength” options. Maximum likelihood trees were inferred using FastTree (122) with the “-gtr” option. The trees were visualized using FigTree. The OTUs of the following results were excluded in the subsequent analyses: (i) the phylogenetic positions of divided sequences were clearly different and (ii) the sequence was extremely long branched. After manual curation, 123 OTUs were excluded leaving 216 OTUs in the final data set (see Table S5 in the supplemental material). To examine the phylogenetic position of these OTUs, we performed an ML analysis. We prepared the 18S data set of almost the entire Chytridiomycota by adding environmental sequences available in GenBank (see Table S6 in the supplemental material) to the reference data set used above (see Table S4) and sequences were aligned and trimmed as above. Subsequently, the curated PacBio OTU sequences were added to this alignment as above. The ML tree was inferred using IQ-TREE 2 with the GTR + F + R6 model selected by ModelFinder. A standard non-parametric bootstrap analysis of 100 replicates was performed. The trees were visualized and edited as above.

## Data Availability

The sequence data obtained in this study were deposited in GenBank under the accession numbers OQ687116–OQ687331 and OQ702805 –OQ702950.

## SUPPLEMENTAL MATERIAL

Data Set S1 mbio.01313-23-s0001.csv OTU table showing the read abundance of PacBio OTUs in each sample.

Figure S1 mbio.01313-23-s0002.pdf Maximum likelihood (ML) tree of 18S rDNA data set.

Table S1 mbio.01313-23-s0003.xlsx List of single cell isolates with sequencing data.

Table S2 mbio.01313-23-s0004.xlsx List of sampling locations.

Table S3 mbio.01313-23-s0005.xlsx List of taxa and environmental sequences used for the phylogenetic analysis of the concatenated data set (18S, 5.8S, 28S rDNA).

Table S4 mbio.01313-23-s0006.xlsx List of sequences of cultures and single cell isolates used for the phylogenetic analysis of 18S rDNA data set.

Table S5 mbio.01313-23-s0007.xlsx List of the curated PacBio OTU sequences.

Table S6 mbio.01313-23-s0008.xlsx List of environmental sequences used for the phylogenetic analysis of 18S rDNA data set.

ASM does not own the copyrights to Supplemental Material that may be linked to, or accessed through, an article. The authors have granted ASM a non-exclusive, world-wide license to publish the Supplemental Material files. Please contact the corresponding author directly for reuse.
