# Supplementary figures and images for: A combined microscopy and single-cell sequencing approach reveals the ecology, morphology, and phylogeny of uncultured lineages of zoosporic fungi
Source: mBio. 2023 Jul 24;14(4):e01313-23. doi: 10.1128/mbio.01313-23 (PMC10470594; doi:10.1128/mbio.01313-23)

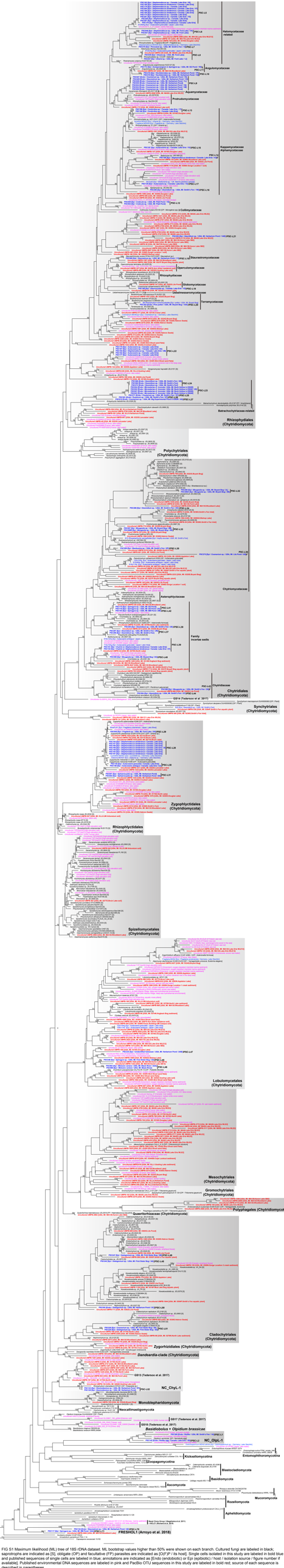

Supplement: Figure S1 — Maximum likelihood (ML) tree of 18S rDNA data set. [file mbio.01313-23-s0002.pdf]
